# Geochemical characterisation of the thermo-mineral waters of Greece

**DOI:** 10.1007/s10653-021-01001-1

**Published:** 2021-06-12

**Authors:** Lorenza Li Vigni, Kyriaki Daskalopoulou, Sergio Calabrese, Konstantinos Kyriakopoulos, Francesco Parello, Filippo Brugnone, Walter D’Alessandro

**Affiliations:** 1 University of Palermo, DiSTeM, via Archirafi 36, Palermo, Italy; 2 University of Potsdam, Institute of Geosciences, Karl-Liebknecht-Str. 24-25, Potsdam Golm, Germany; 3 GeoForschungs Zentrum, Physics of Earthquakes and Volcanoes, Helmholtzstraße 6/7, Potsdam, Germany; 4 Istituto Nazionale Di Geofisica E Vulcanologia, Sezione Di Palermo, via Ugo La Malfa 153, Palermo, Italy; 5 Faculty of Geology and Geoenvironment, National and Kapodistrian University of Athens, Panestimioupolis, Ano Ilissia Greece

**Keywords:** Hydrogeochemistry, Stable isotopes, Carbon dioxide, Geothermometry

## Abstract

Geothermal areas of Greece are located in regions affected by recent volcanism and in continental basins characterised by elevated heat flow. Many of them are found along the coast, and thus, water is often saline due to marine intrusion. In the current study, we present about 300 unpublished and literature data from thermal and cold mineral waters collected along Greece. Samples were analysed for major ions, Li, SiO_2_ and isotopes in water. Measured temperatures range from 6.5 to 98 °C, pH from 1.96 to 11.98, while Total Dissolved Solutes (TDS) from 0.22 to 51 g/L. Waters were subdivided into four main groups: (1) thermal; (2) cold; (3) acidic (pH < 5); and (4) hyperalkaline (pH > 11). On statistical basis, thermal waters were subdivided into subgroups according to both their temperature [warm (< 29 °C), hypothermal (29–48 °C), thermal (48–75 °C) and hyperthermal (> 75 °C)] and TDS [low salinity (< 4 g/L), brackish (4–30 g/L) and saline (> 30 g/L)]. Cold waters were subdivided based on their *p*CO_2_ [low (< 0.05 atm), medium (0.05–0.85 atm) and high (> 0.85 atm)]. *δ*^18^O–H_2_O ranges from − 12.7 to + 2.7‰ versus SMOW, while *δ*^2^H–H_2_O from − 91 to + 12‰ versus SMOW being generally comprised between the Global Meteoric Water Line and the East Mediterranean Meteoric Water Line. Positive *δ*^18^O shifts with respect to the former are mostly related to mixing with seawater, while only for a few samples these shifts point to high-temperature water–rock interaction processes. Only a few thermal waters gave reliable geothermometric estimates, suggesting reservoir temperatures between 80 and 260 °C.

**Supplementary Information:**

The online version contains supplementary material available at 10.1007/s10653-021-01001-1.

## Introduction

In the 1960s, the National Tourism Organisation (NTO, 1966) estimated the number of thermo-mineral springs in Greece at more than 750, with nearly 200 of which found on the islands of the Aegean Sea. Even though about a quarter of these springs is nowadays used for balneotherapeutic purposes, archaeological evidence supports the use of some of them since prehistoric times (Fytikas et al., 1999). In classical times until the end of the Roman Empire, thermo-mineral waters were highly appreciated and many of them were mentioned by poets (Homer, Pindar, Aristophanes), historians (Herodotus, Diodorus Siculus, Plutarch) and geographers (Strabo, Pausanias) (Fytikas et al., 1999). At the same time, the first theories regarding their origin were developed (Plato, Aristotle) and their medical use was emphasised (Hippocrates). It is worth mentioning that, during this period, many thermo-mineral springs were considered sacred and were dedicated either to the Nymphs or to Asclepius, the God of Medicine. Their religious significance continued until Christian times. Then, this dedication shifted either to Virgin Mary or to Agioi Anargyroi, with the latter being saints known for offering their medical services without reward (Håland, 2009). Their balneotherapeutic use declined under the Byzantine Empire and grew again under the Ottoman Empire, but it was not before the end of the twentieth century that other uses (heat and energy production, industrial use of CO_2_, etc.) were applied (Fytikas, 1988). In fact, Fytikas et al. (2005) documented that in the early 70’s the Institute of Mineral and Geological Exploration (IGME) used for the first time the thermal water for greenhouse heating. However, the great development in geothermal field arrived in 1981 when Greece entered the European Union, and many research projects were funded by the European Commission.

Greece, located in a geodynamically active area of the Eastern Mediterranean, is characterised by widespread geothermal resources closely linked to the geology of the country (Papachristou et al., 2019). Its puzzling geodynamic regime contributes to the existence of an elevated terrestrial heat flow, which results in several geothermal fields containing both low and intermediate temperature fluids (Fytikas & Kolios, 1979). Areas of enhanced heat flow are located in regions affected by Miocene or Quaternary volcanism and in continental basins (Fytikas & Kolios, 1979). Geothermal exploration has found high-enthalpy geothermal fields in Milos and Nisyros islands, in the South Active Aegean Volcanic Arc (SAAVA), and low-medium enthalpy georesources in some Aegean islands, i.e. Chios, Lesvos and Samothraki, and in several sedimentary basins of Northern and Central Greece (Mendrinos et al., 2010). Besides, many of these fields are found along the coast as well as in islands, and thus, thermal waters are often brackish to saline due to marine intrusion into the costal aquifer (Lambrakis & Kallergis, 2005). In fact, according to Minissale et al. (1997), the thermal springs located in the SAAVA are affected by mixing between the local meteoric waters and the Aegean seawater, while a marine component, sometimes evolved due to processes seated in the deep thermal reservoirs, is found also in hot waters emerging along the coast of the continental basins (Duriez et al., 2008).

The complex geodynamic and geological setting of the Aegean territory reflects in a great variety of geochemical compositions for many thermal and cold fluid manifestations (Daskalopoulou et al., 2018a, 2019a; Minissale et al., 1989, 1997). The first scientific investigations regarding the chemical composition of the thermo-mineral waters of Greece appeared soon after the birth of the Modern Greek State in 1830. Landerer (1843) gave the first overview of the whole territory and further nationwide studies appeared in more recent times (Athanasoulis et al., 2009; Lambrakis & Kallergis, 2005; Lambrakis et al., 2014; Pertessis, 1937).

**Fig. 1:**
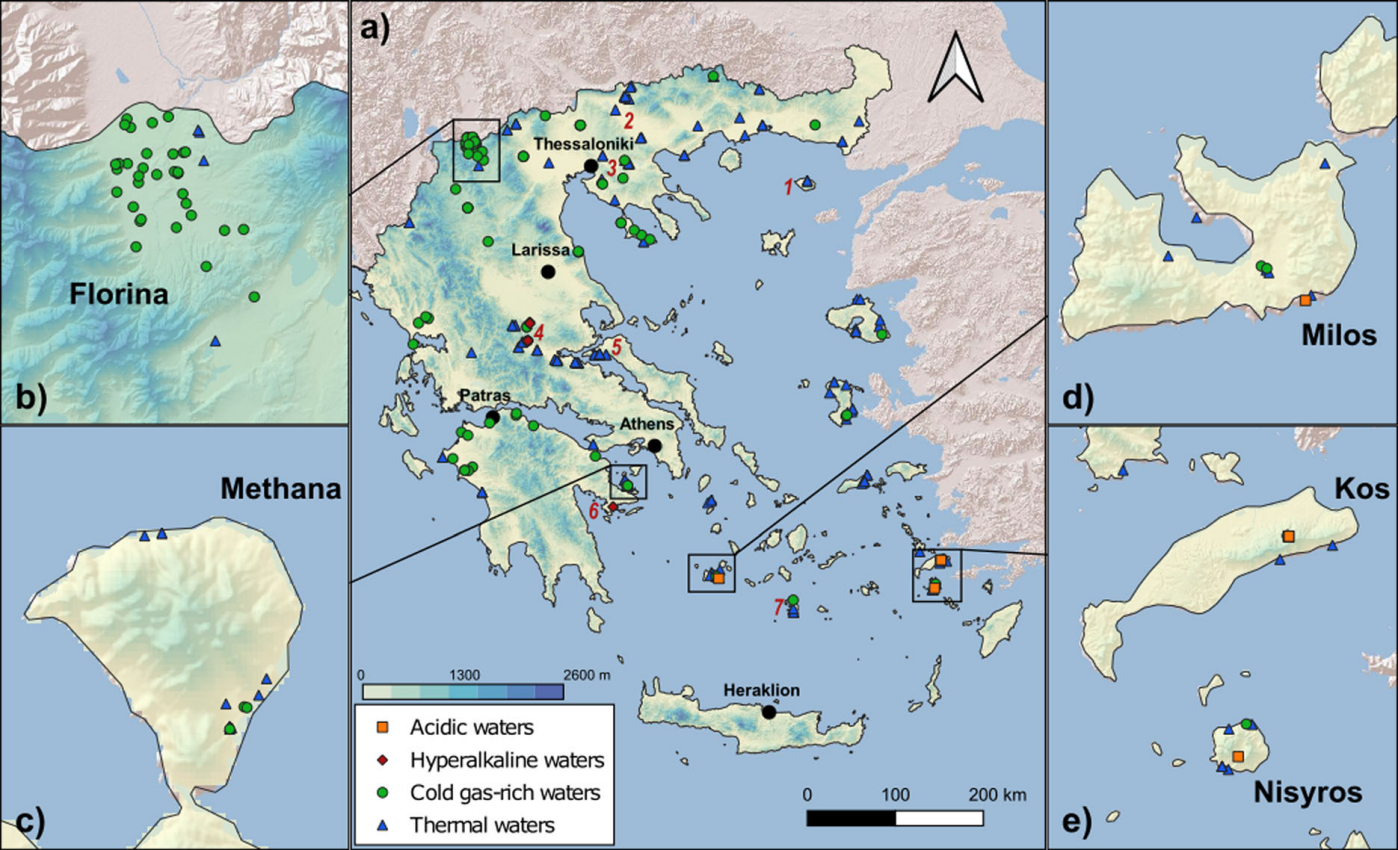
Geographic distribution of the sampling sites. Insets are enlarged areas with high density of sampling sites. *1—Samothraki; 2—Strimon Basin; 3—Migdonia Basin; 4—Othrys and Sperchios Basin; 5—Edipsos; 6—Argolida; 7—Santorini*

The scope of this study is to present a large dataset on the chemical and isotopic composition of the thermo-mineral waters of the whole Greek country and discuss their properties in the framework of the geological context of the area. To this aim, we discussed the results of 285 water samples collected from October 2004 to March 2020 (Fig. 1) and analysed for their major, minor and trace constituents and the isotopic composition of water. About one third of the results were previously published (D'Alessandro et al., 2008, 2014, 2017, 2018, Li Vigni et al., 2021; Papachristou et al., 2014) and are here discussed together with the unpublished data to present a more or less complete picture of the whole country. Although spanning over a long time period, the same sampling and analytical methods were applied increasing the internal consistency of the dataset.

## Study area

The complex geodynamic setting of the Hellenic territory classifies it in the most tectonically and seismically active areas of the world (e.g. Le Pichon et al., 2001; Taymaz et al., 1991; Tsokas & Hansen, 1997). This regime is dominated by three large-scale tectonic structures: (1) the active retreat of the northward subduction of African plate beneath the Eurasian at a rate of 4–6 mm/a (McClusky et al., 2000) forming the back-arc Aegean area; (2) the mostly N–S oriented crustal extension (Mercier, 1981); and (3) the westward motion of Anatolian plate along the strike-slip North Anatolian Fault (NAF) (Pavlides & Caputo, 2004). It is worth noting that the Greek region is the result of the intense collision of several microplates (Aegean, Anatolian and Apulian plate) that took place during the Alpine orogenesis since the Upper Cretaceous involving the subduction of Tethyan Ocean (van Hinsbergen et al., 2005). The volcanism of the area is located in the southern Aegean Sea, while the SAAVA comprises magma of calc-alkaline to shoshonitic suite and signs of crustal contamination (Pe-Piper & Piper, 2006).

**Fig. 2:**
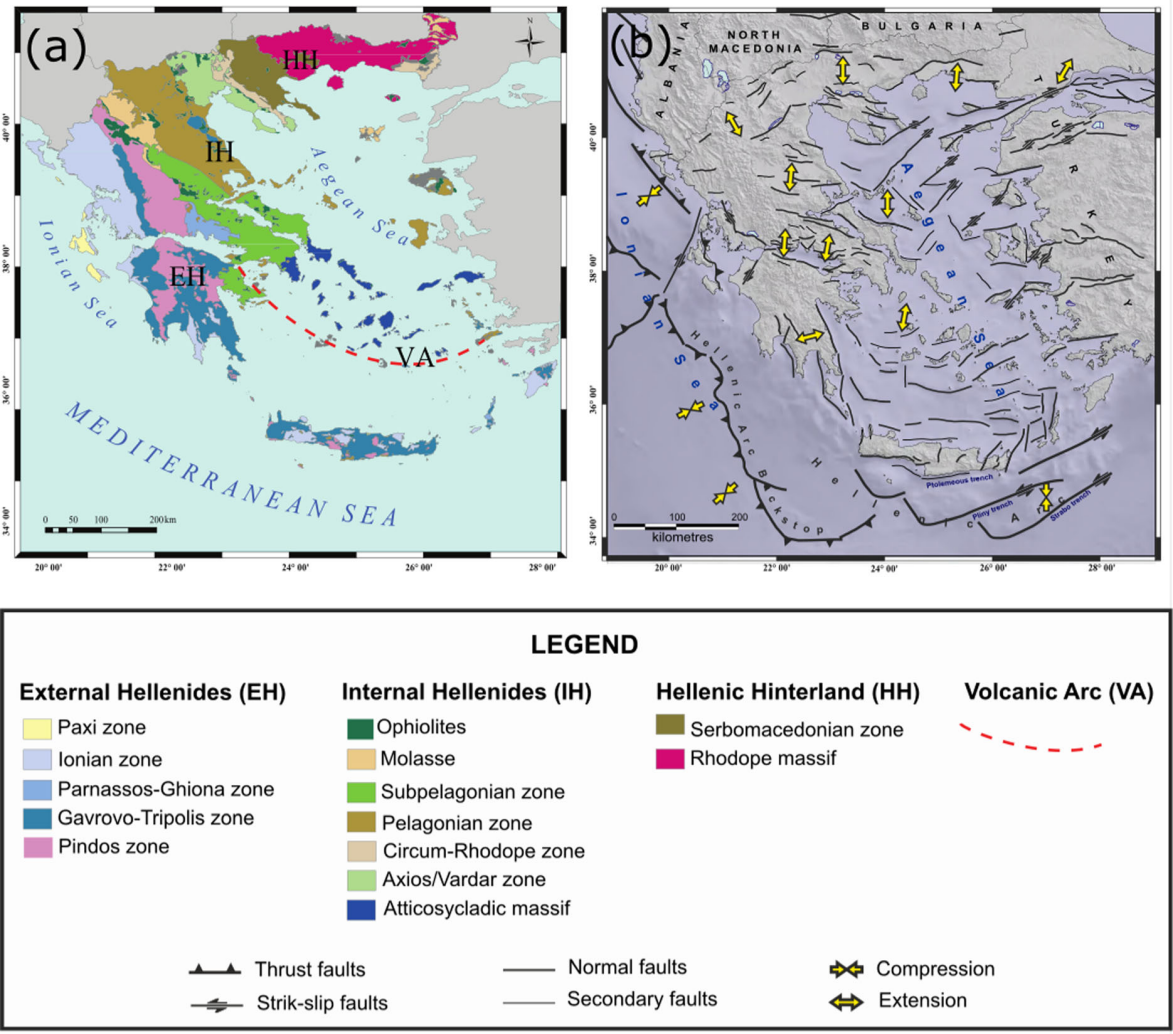
Sketches illustrating the complex geodynamic situation of Greece. **a** Main geologic subdivisions (after Mountrakis, 1985): HH = Hellenic Hinterland; IH = Internal Hellenides; EH = External Hellenides; VA = Volcanic Arc; **b** map of the major tectonic structures and the current horizontal stress field main axes (Pavlides et al., 2010)

Based on the aforementioned complex tectonic setting and the prevailing geological formations, Mountrakis (1985, 2010) divided Greece into structural isopic zones, which from west to the east are:

1. External Hellenides (EH): correspond to a neritic continental sea depositional environment and consist of the Parnassos, Gavrovo-Tripolis, Ionian and Paxos zones. It is worth mentioning that during the Middle-Upper Jurassic, the Ionian zone bearded an intracontinental basin with pelagic sediments. According to Doutsos et al. (2006), three major rift structures occurred during Mesozoic within the eastern margin of the Apulian continent that were reactivated in the Tertiary by forming intracontinental thrusts;
2. Internal Hellenides (IH): express various environments and consist of the Pelagonian, Subpelagonian, Attico-cycladic, Circum-Rhodope and Vardar zones. Depending on their geographical position, they are characterised by obducted ophiolites and deep-sea sediments, neritic sediments, volcanoclastic and sea deposits or flysch. Neritic sediments prevail in the Pelagonian zone, which is considered to be a fragment of the Cimmerian microcontinent, while obducted ophiolites are the most characteristic lithological unit of the Subpelagonian zone. The latter is thought to be the continental slope of the Cimmerian continent towards the ocean, whose sedimentary remnants form the Pindos zone. Both zones consist of sea deposits and appear a progressively deepening sea towards the west. Similar to the Pelagonian, also the Attico-Cycladic zone is envisaged as a continental fragment with undergone neritic sedimentation. Alpidic lithostratigraphic succession bearing volcanoclastic and sea deposits ending up in deep-sea sediments westwards, and flysch are the lithologies characterising the Circum-Rhodope zone. The Vardar zone corresponds to the ocean of Tethys and is characterised by the presence of deep-sea sediments and obducted ophiolites;
3. Hellenic Hinterland (HH): comprises a Precambrian-Silurian continental crust affected by Alpidic metamorphism and consists of the Rhodope and Serbomacedonian Massifs. Crystalline rocks are the main lithology for both zones, while neritic deposits and Late Eocene–Early Oligocene granitoid intrusions are present (Fig. 2a).

From a hydrogeological point of view, the outcropping lithologies in Greece can be subdivided in three major groups. The first is the porous aquifers comprising mainly Quaternary and Neogene sediments (Daskalaki & Voudouris, 2008). These are mostly found within subsident extensional basins and cover about 30% of Greece. The second group comprises all karstic aquifers developed both in limestone of the sedimentary sequences and in marble within metamorphic complexes (Kallioras & Marinos, 2015). The former crop out mainly in central, western and southern Greece and the latter in the northern part of the country, altogether covering about 35% of the whole area. The third group comprises all the remaining lithologies characterised by low permeability or being impermeable (flysch, clays, most metamorphic rocks, volcanites, ophiolites etc.).

Geology along with volcanism and tectonics favoured the existence of many thermal manifestations and anomalous degassing areas (Daskalopoulou et al., 2019a). The elevated heat flow resulted in numerous geothermal fields from low to high enthalpy (Andritsos et al., 2015; Fytikas & Kolios, 1979), while the extensional tectonics (Fig. 2b) affected crustal thinning contributing to fault formation (Grigoriadis et al., 2016) and thus to the ascent of fluids. The elevated heat flow values noticed in the northern part of Greece were associated with the existence of a “first phase” volcanic arc (Fytikas et al., 1984; Vougioukalakis et al., 2004).

## Materials and methods

The physico-chemical parameters (temperature, pH, Eh and Electric Conductivity (EC)) were measured in situ with portable instruments. The total alkalinity was determined by titration with 0.1 N HCl on unfiltered samples (expressed as mgHCO_3_^−^/L). Water samples were filtered (0.45 μm MF-Millipore cellulose acetate filters) and stored in HDPE bottles, while the aliquot for determination of cation contents was acidified with ultrapure concentrated HNO_3_. Analyses of the water chemistry and the isotopic composition were carried out at the laboratories of Istituto Nazionale di Geofisica e Vulcanologia of Palermo (INGV-Pa).

Water chemistry was analysed using standard methods (APHA et al., 2005): major cations (Na, K, Mg, Ca) and major anions (F, Cl, NO_3_, SO_4_) were determined by Ionic Chromatography (ICS-1100, Dionex), Si was determined by Inductively Coupled Plasma—Optical Emission Spectrometry (ICP-OES; Yobin Ultima). Spectrophotometric methods were used for the determination of NH_4_ (Berthelot’s reaction). Lithium was determined by Inductively Coupled Plasma—Mass Spectrometry (ICP-MS; Agilent) as well as Mg and K, when found below detection limits in Ionic Chromatography. For all these analytical methodologies, precision was always better than ± 3%. Speciation of waters and Saturation Index (SI) of main mineral phases for each water sample and the calculation of theoretical *p*CO_2_ for cold gas-rich samples were obtained using the aqueous speciation PHREEQC software (Parkhurst & Appelo, 1999).

Ionic balance (%) was calculated with the formula:

1 $$\{ (\sum cat + \sum an)/[(\sum cat - \sum an)/2]\} \times100$$

where cat are Na^+^, K^+^, Mg^2+^ and Ca^2+^ and an Cl^−^, NO_3_^−^, SO_4_^2−^ and total alkalinity all expressed as meq/L.

TDS (Total Dissolved Solutes) expressed in g/L is here intended as the sum of all major anions (F^−^, Cl^−^, NO_3_^−^, SO_4_^2−^ and alkalinity as HCO_3_^−^) and cations (Na^+^, K^+^, Mg^2+^ and Ca^2+^) plus SiO_2_.

The oxygen and hydrogen isotopic compositions of waters were determined by using, respectively, Analytical Precision AP 2003 and Finnigan MAT Delta Plus IR Mass Spectrometry on unfiltered samples. Results are expressed in delta notation (‰) with respect to the international standard V-SMOW (Vienna Standard Mean Ocean Water). The uncertainties are ± 0.1‰ for *δ*^18^O and ± 1‰ for *δ*^2^H (± 1 *σ*).

Geothermometric estimations were obtained for some selected samples with the software Solute Geothermometers (SolGeo) that includes 35 geothermometric equations (Verma et al., 2008).

## Results

### Water geochemistry

The study area from where the waters were collected is characterised by a great diversity of lithologies and geodynamic environments, which is reflected in a large variety of measured physical–chemical parameters and chemical compositions (Supplementary Material—Table S1). Temperatures measured at spring outlet range from 6.5 to 98 °C, while pH varies from 1.96 to 11.98. It is worth noting that the great majority of the samples is delimited in pH values between 5.5 and 9, while only few springs show either low to very low pH (< 5) or very high pH values (> 11). TDS concentrations range from 0.22 to 51 g/L. Based on the aforementioned parameters, the sampled waters were divided into cold (< 23 °C) and thermal (> 23 °C) waters, with the former being subdivided according to their *p*CO_2_ values and the latter according to their combined temperature values and TDS concentrations (low salinity, brackish and saline). Waters characterised by either very low or very high pH were considered as extra categories. The ionic balance of the cold and thermal waters is generally within the acceptable range of ± 10%. Only three samples of each of these groups exceed such limit (3.2% of the cold and 1.8% of the thermal waters). On the contrary, both acid (80%) and hyperalkaline (16.7%) waters show often strong imbalances. These strong imbalances are not due, as normally considered, to analytical errors. They are mostly due to the presence of less common ionic species not considered in the calculation of the ionic balance. These are OH^−^ in the case of hyperalkaline waters, H^+^, NH_4_^+^, ionic species of Fe, Mn, Al, Sr, Ba in the case of acidic waters, and ionic species of S(-II) and of organic molecules in the case of reduced waters. Almost all of the waters with strong imbalances here considered fall within one of these categories.

**Fig. 3:**
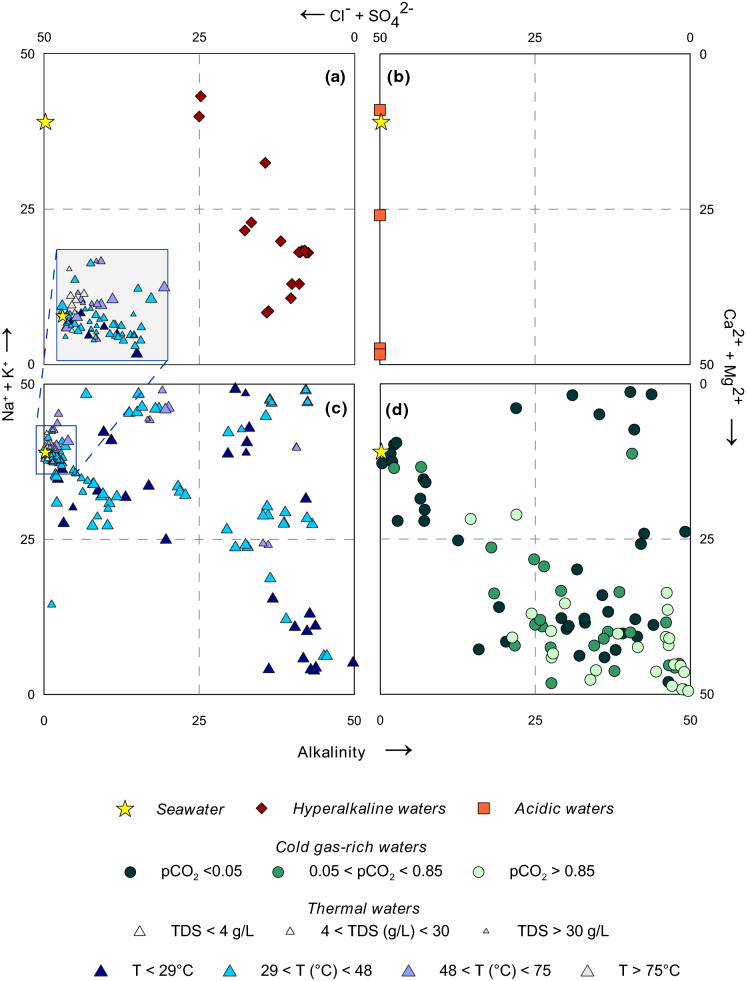
Langelier–Ludwig classification diagram for **a** hyperalkaline, **b** acidic, **c** thermal and **d** cold gas-rich waters. The yellow star represents the composition of seawater

To better discriminate the different geochemical characteristics of the different groups, the data were plotted in a Langelier–Ludwig (1942) classification diagram (Fig. 3) and are described in detail below.

#### Hyperalkaline waters

The hyperalkaline waters are found in the Argolida ophiolites (Peloponnese) and the ophiolitic complex of Othrys (Central Greece). Samples of this group are characterised by very high pH values (11.17 and 11.98) and low salinity (TDS < 0.63 g/L). Temperature ranges from 17.4 to 27.7 °C, while alkalinity is mainly accounted by OH^−^ ions. According to Barnes et al. (1967), they can be classified as Ca–OH waters (Fig. 3a).

#### Acidic waters

The acidic waters are located in the islands of SAAVA, i.e. Kos (Kokkino Nero and Aspro Nero springs), Nisyros (Stefanos) and Milos (Paleochori). They are characterised by low pH values and a wide range of temperatures, which varies from 1.96 to 4.70 and from 13.9 to 98 °C, respectively. It should be noted that the lowest pH (1.98) and the highest temperature (98 °C) values were documented in one water sample of Stefanos crater (Nisyros). These samples are characterised by CaMg–SO_4_ composition (Fig. 3b). Exception is Paleochori (Milos), which falls close to the Aegean seawater point and presents the highest TDS content (51.2 g/L).

#### Thermal waters

Samples from this group were collected in hydrothermal fields located along the SAAVA and in continental basins. The diversity of the settings in which they were collected results in a wide range of temperature (from 23.2 to 95.5 °C), pH (from 5.73 and 10.01) and salinity (from 0.3 to 43.2 g/L) values. Based on their temperatures and TDS content, they are subdivided into different classes (Fig. 3c); this subdivision was made following the method of Sinclair (1974) to identify statistically different populations. Three populations were detected based on TDS, while four populations were identified considering the measured temperatures (Fig. 3c). According to their water compositions, they fall into three of the quadrants of the Langelier–Ludwig diagram (Fig. 3c): (1) CaMg-HCO_3_ and (2) Na–HCO_3_ water-types, which include mainly low salinity samples (< 4 g/L) and few intermediate salinity samples (4 < g/L < 30), with temperatures almost below 48 °C; (3) NaCl composition, which includes high temperature and salinity samples with compositions that fall mostly close to the seawater point.

#### Cold gas-rich waters

The cold water samples are characterised by the presence of high levels of geogenic gases found in free and/or dissolved phase. Temperature ranges from 8.8 to 23 °C, pH between 5.20 and 8.72, while salinity shows a wide spectrum of values in terms of TDS (from 0.31 to 30.1 g/L). Based on their CO_2_ content calculated as *p*CO_2_ using the speciation software PHREEQC (Parkhurst & Appelo, 1999), they were divided into three groups; the groups were identified with the method proposed by Sinclair (1974). After plotting the samples in the Langelier–Ludwig diagram (Fig. 3d), it is noticed that the great majority of the waters with *p*CO_2_ > 0.05 falls in the CaMg–HCO_3_ field, while samples characterised by low *p*CO_2_ (< 0.05) are scattered in all the sectors of the diagram.

### Isotopic composition

**Fig. 4:**
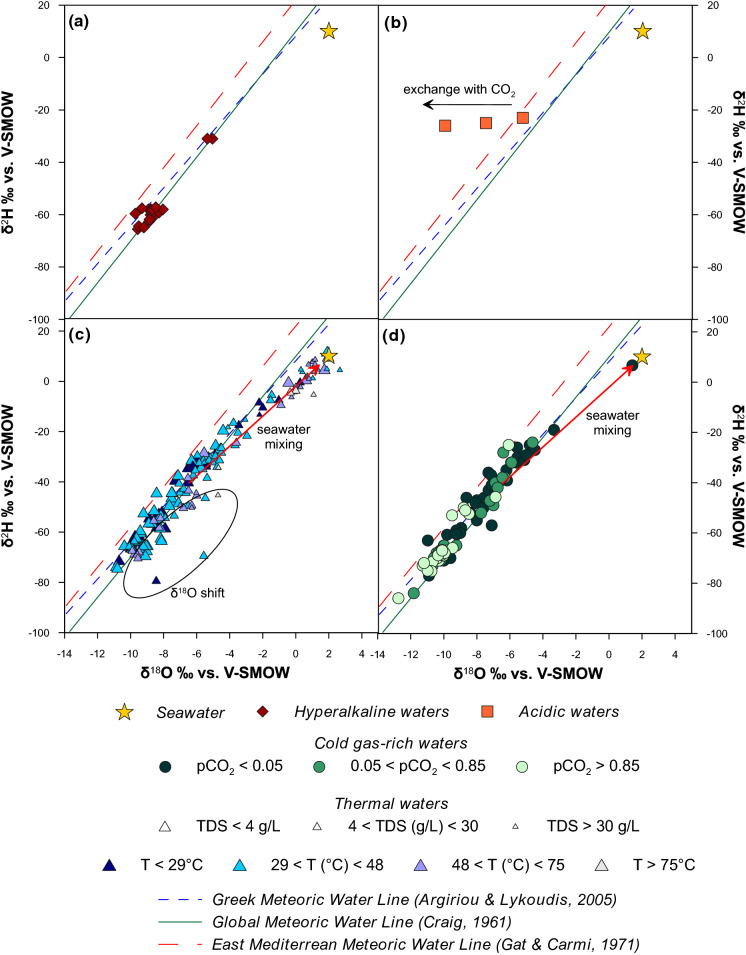
*δ*^2^H versus *δ*^18^O binary diagrams for the sampled waters

The isotopic composition of the collected waters ranges from − 2.7 to + 2.7‰ for *δ*^18^O and from − 91 to + 12‰ for *δ*^2^H. In the *δ*^2^H–*δ*^18^O diagram (Fig. 4), the majority of the waters fall between the Global Meteoric Water Line (Craig, 1961: GMWL *δ*^2^H = 8 × *δ*^18^O + 10) and the East Mediterranean Meteoric Water Line (Gat & Carmi, 1971: EMMWL *δ*^2^H = 8 × *δ*^18^O + 22). A Local Meteoric Water Line defined by Argiriou and Lykoudis (2006) (LMWL *δ*^2^H = 7.24 × *δ*^18^O + 8.2) has been also plotted. Such LMWL has been obtained by the authors including all published isotope data on rainwater collected in Greece in the period from 1960 to 2003 (Argiriou & Lykoudis, 2006). Hyperalkaline (Fig. 4a) and most of the thermal and cold waters (Fig. 4c, d) follow the LMWL of Greece. This suggests that the water samples have a meteoric origin, whereas acidic waters show a negative shift for *δ*^18^O (Fig. 4b).

Some thermal and cold waters are aligned with Aegean seawater point (Fig. 4c, d), indicating a mixing between the meteoric water and seawater. As marked in Fig. 4c and 4, many water samples show a positive *δ*^18^O shift. In the case of thermal waters this could indicate isotope exchange due to water–rock interaction at higher temperatures (Clayton et al., 1972), while in the case of cold waters it may be justified by evaporation processes.

**Fig. 5:**
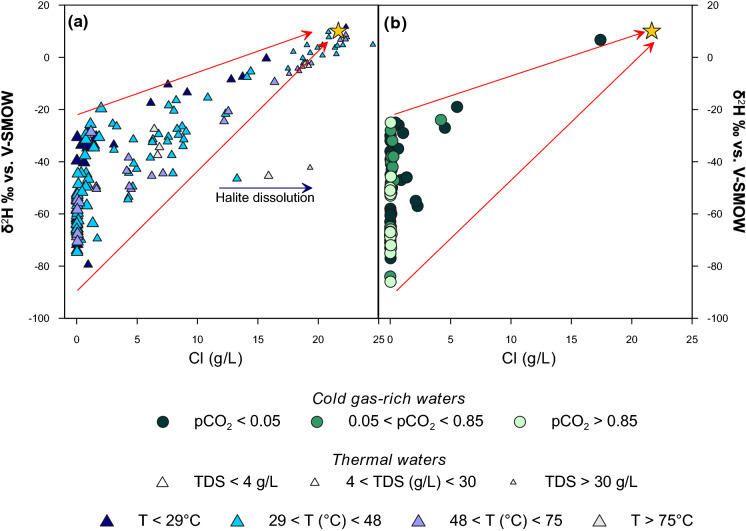
*δ*^2^H versus chloride binary diagrams for the sampled waters. Red arrows show mixing lines between the highest and lowest average isotopic value of rainwater measured in Greece (Argiriou & Lykoudis, 2006)

The effect of seawater contamination can be observed in the *δ*^2^H versus Cl binary diagram (Fig. 5). Only few cold gas-rich waters show important seawater admixing (Fig. 5b), while this process is very widespread for the thermal waters (Fig. 5a). Some waters collected in Samothraki Island fall outside the rainwater-seawater mixing lines. For these, dissolution of Halite in Miocene evaporites have been invoked (Dotsika, 2012).

## Discussion

### Hyperalkaline waters

The ophiolitic sequences comprise widespread products derived from the hydration of ultramafic rocks, in which olivines and pyroxenes are altered to serpentine-group minerals (Evans et al., 2013). Typical features of waters circulating in serpentinised ultrabasic rocks are elevated pH values (> 10), Ca–OH composition with very low concentrations of Mg^2+^ and total dissolved carbon (TDC) mainly present as CO_3_^2−^ (Bruni et al., 2002). According to Barnes et al. (1967), two types of waters can be recognised in aquifers hosted in ophiolitic rocks: (1) MgCaHCO_3_ waters with pH generally < 10 and (2) hyperalkaline Ca–OH waters. The former represents the early stage of interaction between the ophiolites and meteoric waters into shallow aquifers (Cipolli et al., 2004), while the latter represents its evolution in deep serpentinised ultramafic aquifers under reducing environment (Bruni et al., 2002). Samples of Argolida ophiolites (Peloponnese) are represented by the Agioi Anargyroi Springs, which are characterised by enhanced pH values (up to 11.98) and Ca–OH composition (D’Alessandro et al. 2018). On the other hand, samples of Othrys (Central Greece) are characterised by many hyperalkaline springs (pH > 11; Ekkara and Archani), which are classified as Ca–OH-type waters (D’Alessandro et al. 2014; Li Vigni et al., 2021).

### Acidic waters

Samples of this group present characteristics similar to acidic waters as described in Giggenbach (1988). These waters can be distinguished in three different subgroups each one belonging to a different island of the SAAVA.

Samples collected at Kos Island are characterised by acid sulfate composition, likely related to the addition of CO_2_- and H_2_S-rich hydrothermal gases to groundwater (Giggenbach, 1988), and exhibit high concentrations of Ca^2+^ (up to 508 mg/L) and SO_4_^2−^ (up to 2,774 mg/L). These springs are found within an area of strong geogenic soil degassing (Daskalopoulou et al., 2019b). The abundant deep hydrothermal H_2_S gas dissolves in the shallow aquifer and is converted into H_2_SO_4_ by oxidation with atmospheric oxygen dissolved in the meteoric recharge (Nordstrom et al., 2009). Such process increases the sulfate content and lowers the pH of the water. The isotopic composition shows a negative shift of *δ*^18^O (up to about 5‰ units) with respect to the local meteoric water line (Fig. 4b). The effect can be the result of isotope exchange with CO_2_ (Karolytè et al., 2017) at low temperature favoured by the high gas/water ratio.

The sample of Stefanos crater (Nisyros) is characterised by an elevated temperature (98 °C), very low pH value (1.98) and an acidic sulfate composition. It was collected in a boiling pool of the hydrothermal explosion crater of Stefanos within the Lakki caldera. The crater is characterised by strong fumarolic activity (Marini & Fiebig, 2005), mainly concentrated along the rim. An area of about 1000 m^2^ with many tens of boiling pools is present in the middle of the crater, the water of which probably results from the mixing of condensing fumarolic vapour and meteoric-derived shallow groundwater. In the summer dry period, the pools’ level falls to about 1 m depth, while in winter, after heavy rainfall, the entire bottom of the crater can be covered by rainwater, forming an ephemeral lake. The collected sample has the typical steam-heated water composition (Nordstrom et al., 2009) with high concentrations of SO_4_^2−^, low pH and high temperature. Sulfate is almost the only anion balanced by a lot of cations including Fe, Al and NH_4_^+^ that have concentrations comparable to the major cations.

The Paleochori sample was collected at a small cave on the western end of Paleochori Beach (Milos) and is characterised by high temperature (75 °C) and NaCl water type. The water of the spring comes out with gas bubbles from rocky debris and gets mixed with seawater, when the sea is rough. Its chemical composition is similar to marine water, indicating that, even if the sea is calm, contamination by seawater occurs within the shallowest part of the hydrological circuit. The area is characterised by widespread degassing both onshore as diffuse degassing from the beach and underwater as bubbling hot water (up to 120 °C) springs (Daskalopoulou et al., 2018b).

### Cold gas-rich waters

As mentioned in Par. 4.3, cold mineral waters have been subdivided into three statistically different populations based on their *p*CO_2_ (< 0.05—from 0.05 to 0.85 and > 0.85 atm), with the lowest value almost corresponding to the highest limit of organic derived soil CO_2_ contribution to groundwater (Chiodini et al., 1995). Therefore, values above this limit indicate a geogenic CO_2_ contribution. Recent studies (Daskalopoulou et al., 2018a, 2019a) evidenced that Greece, being a geodynamically active region, is a territory of extensive geogenic degassing. Most of the degassing activity in Greece is associated to thermal anomalies of variable intensity, but especially in Northern Greece the two phenomena are sometimes unrelated. In this area, several cold CO_2_-rich mineral waters are known and many of them are appreciated as natural soda waters suitable for human consumption, with some of them being distributed in the whole country (i.e. Doumbia, Souroti and Xino Nero).

Most of the CO_2_-rich groundwater samples were collected in Florina Basin. This is one of the intramontane basins within the Hellenide Orogen, formed by the extensional tectonic regime of the area starting from the Late Miocene (Pavlides & Mountrakis, 1987). The thick and impermeable sedimentary sequence of Florina basin favoured the existence of many CO_2_ reservoirs (Karakatsanis et al., 2007). Some of these are industrially exploited by the Air Liquide Greece Company for production of dry ice and filling of pressurised gas bottles. The estimated industrial CO_2_ extraction is ~ 30,000 t/a (Pearce, 2004). However, these reservoirs are leaky, thus CO_2_ rises up, mainly through faults, reaching directly the atmosphere or being dissolved in great quantities in the shallow unconfined aquifers. The water extracted from many of the shallow wells (< 100 m) dug or drilled in these aquifers separates a free gas phase due to the high concentration in CO_2_ (D’Alessandro et al., 2011).

The equilibrium of carbonate species is regulated mainly by sources and sinks within the hydrologic circuit. The main sources are the deep-derived geogenic CO_2_ dissolution and/or the dissolution of carbonate rock of the aquifers. Losses depend mainly on CO_2_ exsolution due to the separation of a free gas phase as water pressure within the aquifer decreases in the shallower levels and/or on precipitation of carbonate minerals due to oversaturation. The majority of samples with high *p*CO_2_ are characterised by Ca–HCO_3_ composition. The dissolution of CO_2_ strongly dominates the chemical evolution of these waters increasing their aggressiveness with respect to the aquifer’s rocks.

**Fig. 6:**
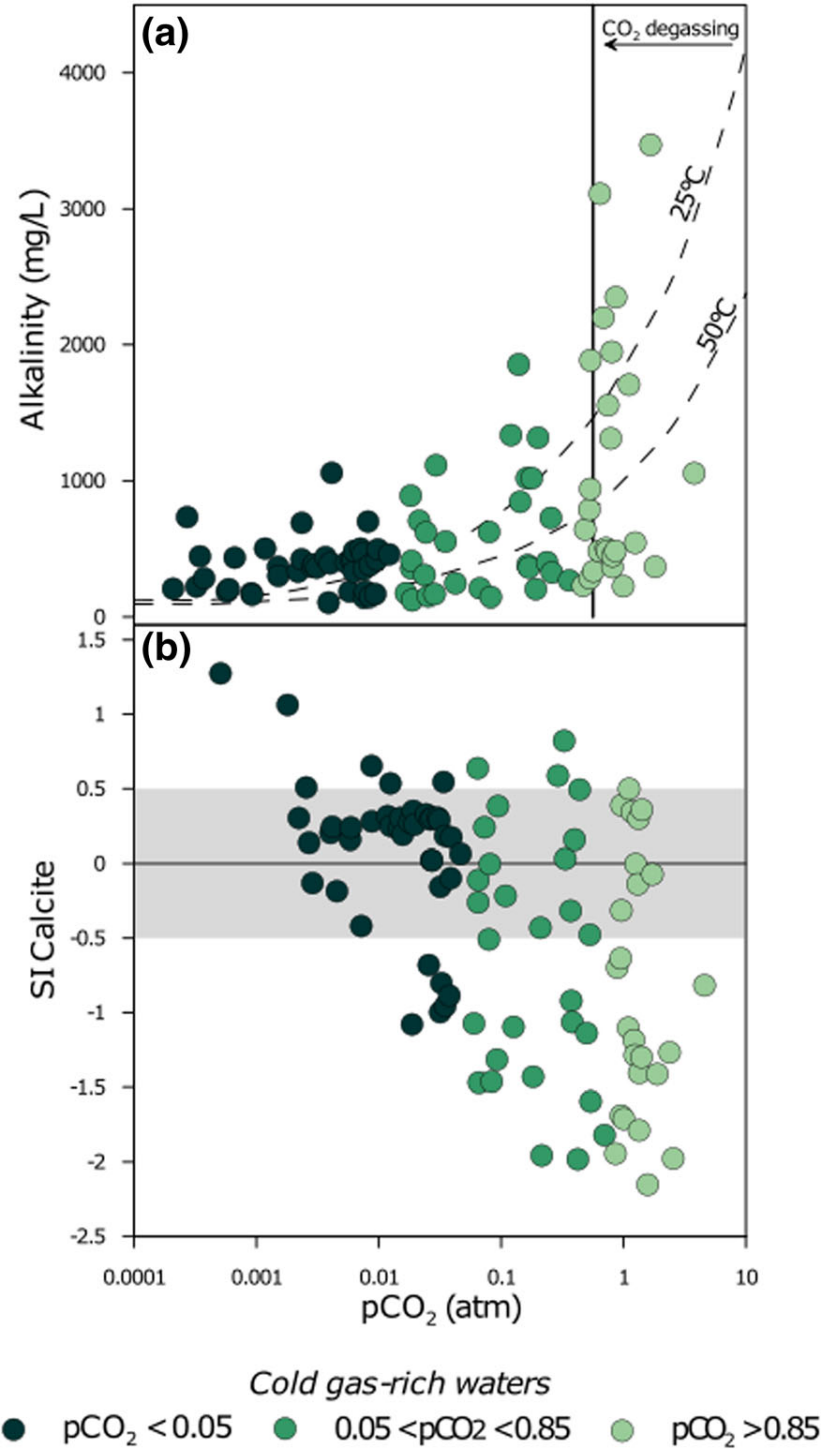
CO_2_ partial pressure versus alkalinity (**a**) and versus calcite saturation index (**b**) of the cold gas-rich waters. Dashed lines in (**a**) are concentrations of alkalinity (expressed as mg/L of HCO_3_^−^) at the given CO_2_ partial pressure at 25 and 50 °C. The grey shaded area in (**b**) comprises values (± 0.5) considered at saturation for the given solid phase

As a consequence, bicarbonate represents generally more than 70% by weight of their TDS, and pH is slightly acidic (5.2–6.9). Due to the high *p*CO_2_ values (Fig. 6a), in this group of waters, saturation or oversaturation in carbonate minerals (Calcite, Aragonite or Dolomite) is rarely achieved (Fig. 6b). In the intermediate *p*CO_2_ population, only few samples result oversaturated (Fig. 6b). Equilibrium with carbonate minerals is rarely attained in the waters with *p*CO_2_ > 0.05 atm indicating that carbonate dissolution in these waters is not so important as the CO_2_ dissolution. On the contrary, many of the waters with *p*CO_2_ < 0.05 atm are close to equilibrium with the main carbonate minerals. These waters are from sedimentary areas, where the presence of carbonate rocks in their aquifers is ascertained or probable and these rocks control the equilibrium of the dissolved carbonate species. In these waters, the dissolved and/or, if present, the free gas phases are dominated by CH_4_ or N_2_ (Daskalopoulou et al., 2018a, 2019a). Geographically, these waters are mainly distributed in the western part of Greece, where CO_2_ degassing is trivial and hydrocarbon-rich areas are present (Daskalopoulou et al., 2018a, 2019a).

**Fig. 7:**
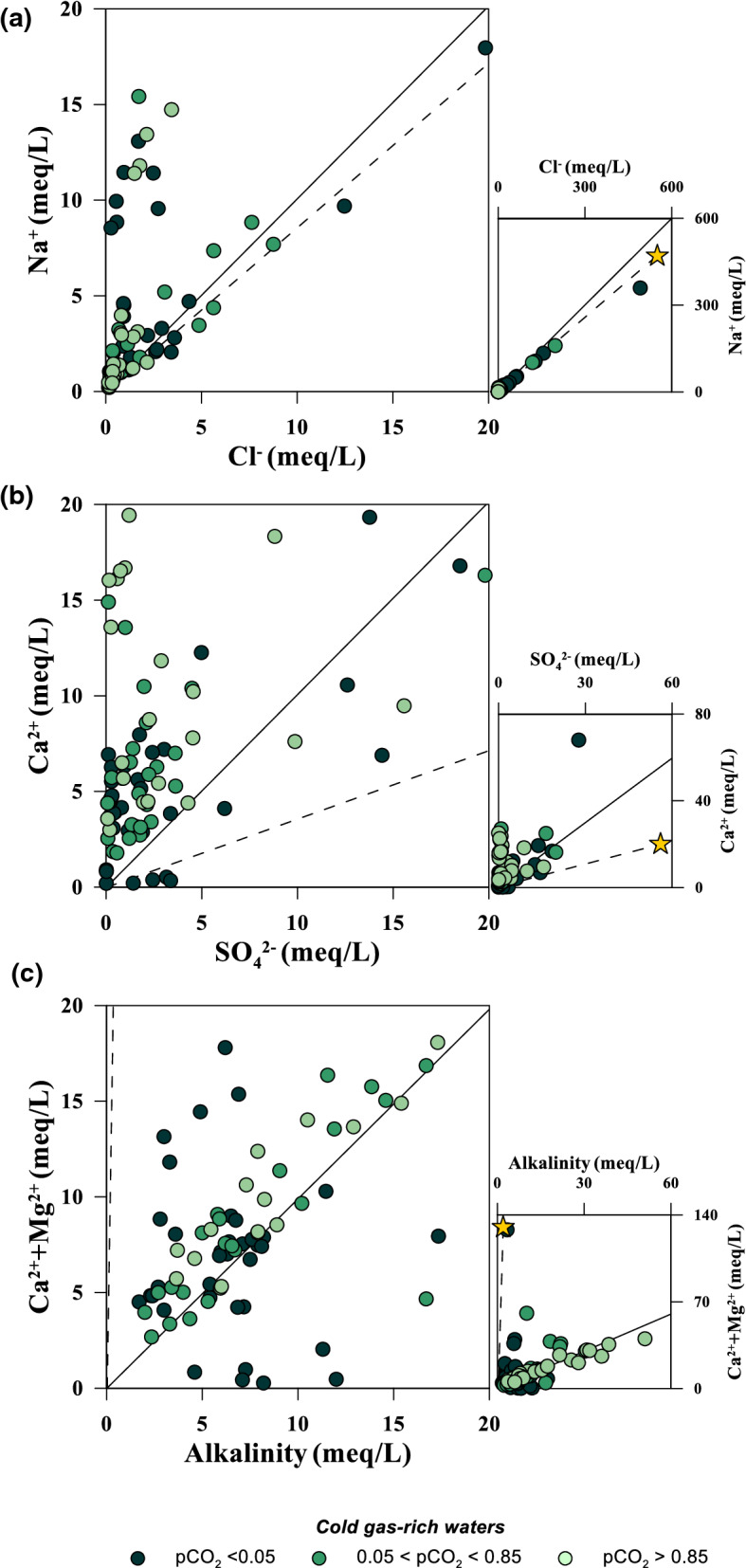
Binary correlation plots for the cold gas-rich waters. **a** Na^+^ versus Cl^−^; **b** Ca^2+^ versus SO_4_^2−^; **c** Ca^2+^ + Mg^2+^ versus alkalinity. Dashed line seawater mixing line, continuous line 1/1 ratio

Only few cold water samples have a NaCl composition, indicating a significant seawater contamination of the aquifers. These are located along the coastlines of continental Greece or on islands. Despite their moderately low temperatures (19.4–20.7 °C) that classify them cold, they have been used in the past as spas and are therefore included in the present inventory. In the Na versus Cl binary diagram (Fig. 7a), they fall along the seawater mixing line. Almost all of the low salinity waters are enriched in Na with respect to the Na/Cl ratio in seawater due to water–rock interaction processes within their aquifers.

Very few water samples plot along the 1/1 ratio line in the Ca^2+^ versus SO_4_^2−^ binary plot (Fig. 7b) suggesting only a sporadic influence of Ca-sulfate minerals dissolution. Most of the waters are strongly enriched in Ca^2+^ confirming the strong influence of Ca-carbonate dissolution. But as we will see for the thermal waters, Ca^2+^ enrichment may occur also in the case of Ca-sulfate minerals dissolution, when the thermodynamic conditions are favourable to sulfate reduction to sulfide. This is probably the case of most waters collected in the western part of Greece, where Triassic evaporitic gypsum is often found in the sedimentary sequences of the area (Rigakis & Karakitsios, 1998). Thermochemical sulfate reduction can be sustained by the presence of hydrocarbons in the same area (Palacas et al., 1986), which promote the sulfide formation (Machel, 2001).

Most of the cold water samples are more or less aligned along the 1/1 ratio in a Ca^2+^  + Mg^2+^ versus alkalinity binary diagram (Fig. 7c) indicating a strong influence of the congruent dissolution of carbonate minerals within their aquifers. Only in few cases the mixing of seawater can be invoked to explain a Ca^2+^  + Mg^2+^ excess with respect to the 1/1 ratio line. On the contrary, most of the deviations from this line may be due to oversaturation of some carbonate species. In this case, the precipitation of a solid phase will virtually enrich either the cations (Ca^2+^ and Mg^2+^) or the anion (HCO_3_^−^) depending on which one is in excess with respect to the 1/1 equivalent ratio. Calcium excess may be favoured by the presence and dissolution of Ca-sulfate minerals, while bicarbonate excess may derive from the dissolution of abundant geogenic CO_2_. The latter process may justify the formation of the alkaline-bicarbonate waters (Fig. 3d).

### Thermal waters

Geologically young regions like the Alpine orogen show a high variability in heat flow with respect to older cratonic areas (Pollack et al., 1993). Greece, which belongs to the Alpine orogen, makes no exception. In the preliminary map of Fytikas and Kolios (1979), heat flow shows values that range from < 30 to > 120 mW/m^2^ (Fig. 10b). Values above the average continental heat flow (65 mW/m^2^—Pollack et al., 1993) are considered as positive anomalies. Looking at the map of Fytikas and Kolios (1979), these anomalous values are mainly found in areas with active or recent (< 10 Ma) intrusive or effusive magmatism (Pe-Piper & Piper, 2002). These areas are also those with the thinnest crust (Grigoriadis et al., 2016) and subject to active extensional tectonics (Pavlides et al., 2010 (Fig. 2b). The highest heat flow anomalies are recorded along the SAAVA. Signs of the increased heat flow are thermal springs and thermal groundwater tapped by drillings. Therefore, it is not a surprise to find the hottest thermal waters in the areas of increased heat flow (Fig. 10b). These thermal waters, together with fumaroles and steaming grounds, are the surface expressions of active geothermal systems. Geothermal exploration proved that two of them, located in the active volcanic systems of Milos and Nisyros, are two-phase high-enthalpy fields suitable for electricity production. Explorative drillings tapped geothermal fluids with temperatures up to 320 and 340 °C, respectively (Chiodini et al., 1993; Liakopoulos et al., 1991). Geothermal energy was exploited for a period by a power plant at Milos until it was stopped by the protests of the inhabitants complaining for the H_2_S released by the extracted geothermal fluids (D’Alessandro et al., 2009). The remaining explored geothermal reservoirs can be defined as hot-water systems (Kaya et al., 2011).

**Fig. 8:**
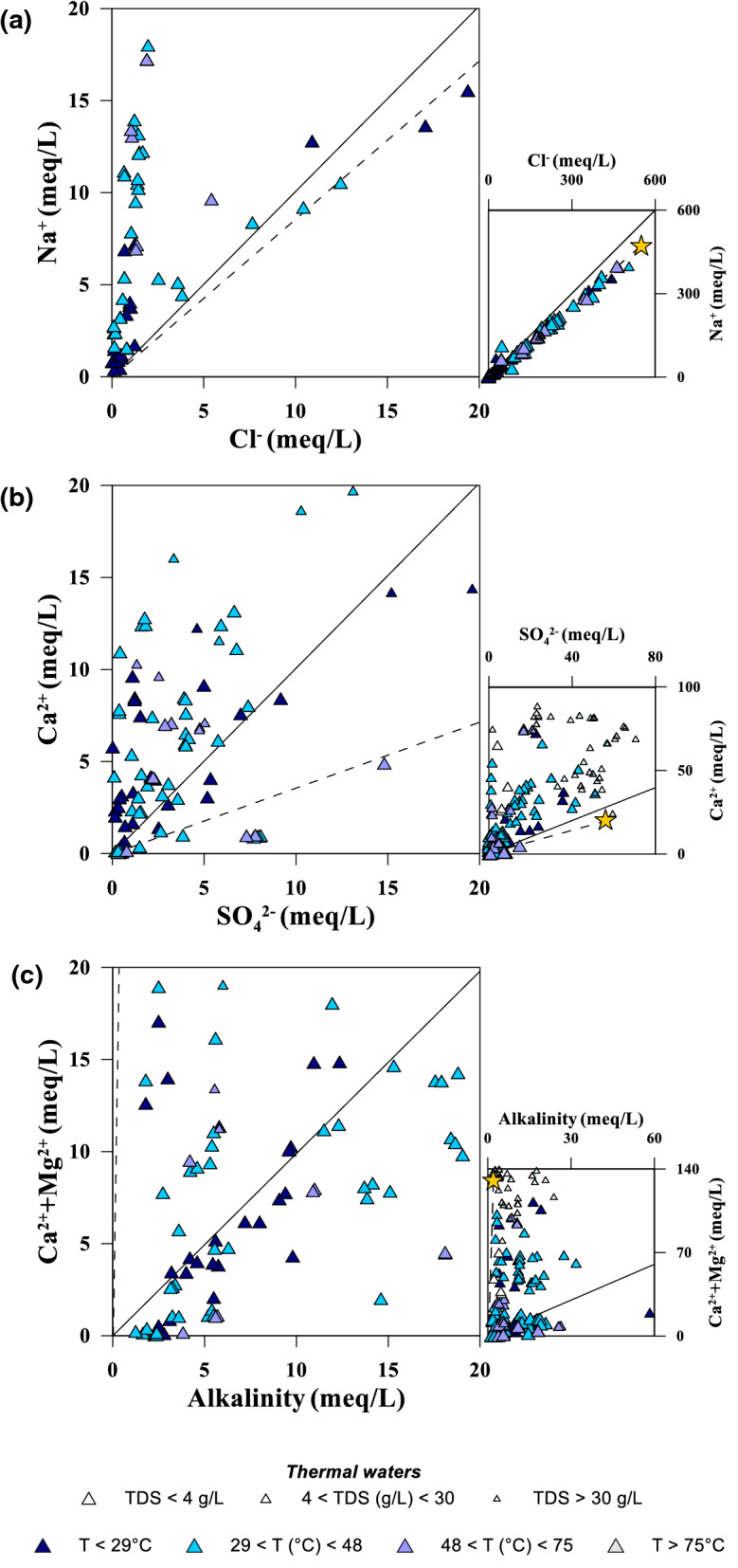
Binary correlation plots for the thermal waters. **a** Na^+^ versus Cl^−^; **b** Ca^2+^ versus SO_4_^2−^; **c** Ca^2+^ + Mg^2+^ versus alkalinity. Dashed line seawater mixing line, continuous line 1/1 ratio

**Fig. 9:**
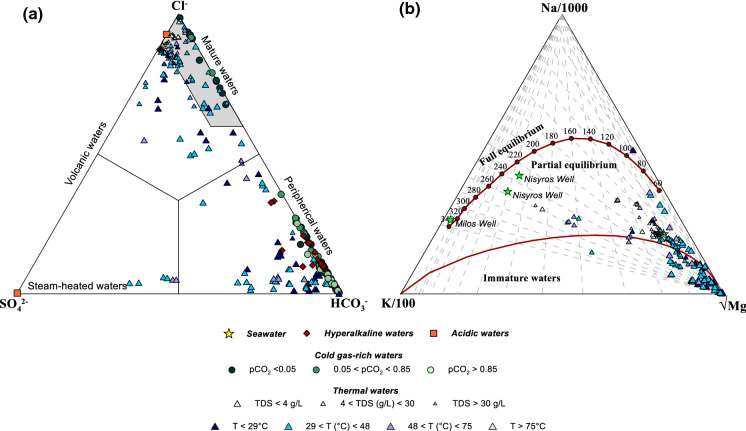
Triangular diagrams of **a** Cl-SO_4_-HCO_3_ (Giggenbach, 1991) and **b** Na–K–Mg (Giggenbach, 1988)

Many of the geothermal systems are found on islands or are on the coast of continental Greece (Fig. 1). Therefore, seawater plays a major role in determining the composition of the waters released by these systems. This can be evidenced both in their chemical composition with Na/Cl ratios very close to that of seawater (Fig. 8a) and in their isotopic composition (Figs. 4 and 5) nicely evidencing a mixing between meteoric and sea water. From these diagrams, it cannot be deduced if seawater acts as a contaminant in the shallowest part of the hydrologic circuit or if it represents a main feeder of the geothermal reservoir. For the high-enthalpy systems of Milos and Nisyros, a strong contribution of seawater to the reservoir has been ascertained from the analyses of the fluids sampled from the explorative boreholes (Chiodini et al., 1993; Liakopoulos et al., 1991). However, a clue of a seawater contribution in several other reservoirs is the fact that many of the samples plotting close to the seawater point are those with the highest temperatures (Figs. 3, 4 and 5). As we will see in par. 5.4.1, these are often waters falling in the field of the partially equilibrated waters in the Giggenbach triangular plot (Fig. 9b).

Many water samples show a very high Na/Cl ratio (Fig. 8a). Almost all come from three graben areas filled by postorogenic sediments mainly deriving from the dismantling of the metamorphic rocks bordering these grabens. These are the Sperchios, Strimon and Migdonia basins. The faults bordering the grabens favoured the formation of hydrothermal systems, while the ubiquitous presence of Na-rich minerals (e.g. albite) in the metamorphic rocks and in the sediments from them derived allowed the formation of Na-rich waters (Meyback, 1987). For the same reasons, similar conclusions may be drawn also for the cold gas-rich waters collected in the Florina basin and some artesian wells along the northern and western coast of Peloponnese (Fig. 7a).

As seen before for the cold waters, also in the case of thermal waters very few samples fall along the line representative of the dissolution of Ca-sulfate minerals (Fig. 8b). The great majority of the waters are enriched in Ca^2+^ that is mostly explainable by the release of this cation by high-temperature water–rock interaction processes. But the sulfate deficit may be, at least partially, explained by the reduction to sulfur and sulfide. While in the high-temperature systems such reduction involves only inorganic reactions in the case of lower temperatures microbiological mediated sulfur reduction is also involved, sometimes presupposing the presence of thermophile microorganisms (Brombach et al., 2003; Chiodini et al., 1998; Gilhooly et al., 2014). The rapidly changing physico-chemical conditions in the shallowest part of the hydrothermal circuit induces rapid changes in the oxidation state of sulfur further complicating the picture in a complex interaction between biotic and abiotic influences on the sulfur cycle (Gilhooly et al., 2014; Marini et al., 2002). This may affect also on other dissolved species like for example methane. Strong isotope fractionation of this gas has been, in fact, attributed to anaerobic methane oxidation involving the microbial reduction of sulfate as electron donor (Daskalopoulou et al., 2018a).

Spas in western Greece are fed by hypothermal or even cold springs. They are generally heated for balneotherapy and considered healthy mainly for their mineral content. Most of them are rich in dissolved sulfide and the exsolved H_2_S can be distinctly smelled. This region corresponds to the thick organic-rich sedimentary sequences of the External Hellenides (Fig. 2a), which are considered the most favourable areas in Greece for hydrocarbon generation (Palacas et al., 1986; Rigakis & Karakitsios, 1998). Thus, in the sampled waters methane is generally the dominant gas both in the dissolved and the free gas phase (Daskalopoulou et al., 2018a, 2019a). This is the Greek region with the greatest crustal thickness and hence the conditions are unfavourable to the uprise of gases and heat from the mantle. Therefore, this area is not prone to the formation of geothermal systems and the slight thermalisation of the waters is mostly the consequence of the deep circulation within regional fault systems. The stratigraphic sequences of the External Hellenides comprise often gypsum-rich Triassic evaporites (Rigakis & Karakitsios, 1998). The presence of sulfates and hydrocarbons favours the formation of H_2_S through either Microbial (MSR) or Thermochemical (TSR) Sulfate Reduction (Machel, 2001). The presence of H_2_S in the water samples in western Greece is mainly related to these processes rather than being produced in active geothermal systems.

Finally, the Ca^2+^  + Mg^2+^ versus alkalinity binary diagram (Fig. 8c) shows that the interaction with carbonate minerals is negligible and evidences two main processes affecting these waters: mixing with seawater and dissolution of geogenic CO_2_.

#### Geothermometry

The ternary diagram Cl-SO_4_-HCO_3_ (Giggenbach, 1991) is used to examine the maturity of waters. Specifically, mature waters are characterised by high Cl content that originates from a deep-hot geothermal system, while SO_4_-type waters usually derive from steam-heated water influenced either by volcanic steam bearing high-temperature HCl or by geothermal steam bearing low-temperature H_2_S (Dolgorjav, 2009; Rezaei et al., 2018). On the other hand, alkaline waters are typically related to samples of meteoric origin. In the Hellenic territory, hyperalkaline waters, as well as the great majority of cold waters and some thermal waters fall in the HCO_3_ field, indicating processes of mixing with the near-surface groundwater during their ascent to the surface (Singh et al., 2015) (Fig. 9a). Enrichment in SO_4_ is present in some thermal (Nymfopetra, Nea Apolonia (1,3,4)) waters. These samples (thermal) present low Cl content, high Na concentration and are characterised by calcite precipitation indicated by the presence of travertines in these areas. The fumarolic samples of Stefanos and Paleochori are plot in the SO_4_-Cl axis, which is typical of volcanic waters, highlighting the impact of the volcanic activity on the samples. Many thermal gases fall in the field of mature waters. These samples may be considered as samples not affected by secondary processes during the ascent of water to the surface that mainly derive from the deep and hot geothermal systems.

In order to evaluate the applicability of conventional geothermometric estimates, the chemical composition of the thermal waters was plotted on a cationic ternary diagram (Giggenbach, 1988) (Fig. 9b). The majority of the samples fall in the immature waters field, and therefore, they have to be considered unsuitable for geothermometric estimations. Much fewer samples plot in the partial equilibrium field, while only one sample (Amplas 2) falls above the full equilibrium line. The latter refers to water in which an abundant CH_4_-rich gas phase was bubbling (Li Vigni et al., 2021). Waters interacting with CH_4_ are often characterised by a strong Mg depletion either because they are mixed with oil field brines (Kharaka & Mariner, 1989) or with hyperalkaline water (Bruni et al., 2002; Cipolli et al., 2004).

The only samples that plot on or very close to the full equilibrium line are those taken from geothermal exploration wells at Milos (well M2—Koutroupis, 1992) and Nisyros (well N_2_—Koutroupis, 1992; Chiodini et al., 1993). The estimated temperatures nearly correspond to that measured at Milos (318 °C—Liakopoulos et al., 1991) or are not so far from that measured at Nisyros (290 °C—Chiodini et al., 1993). In the latter case, Chiodini et al. (1993) hypothesise a contamination by the seawater used to prepare the drilling mud.

**Fig. 10:**
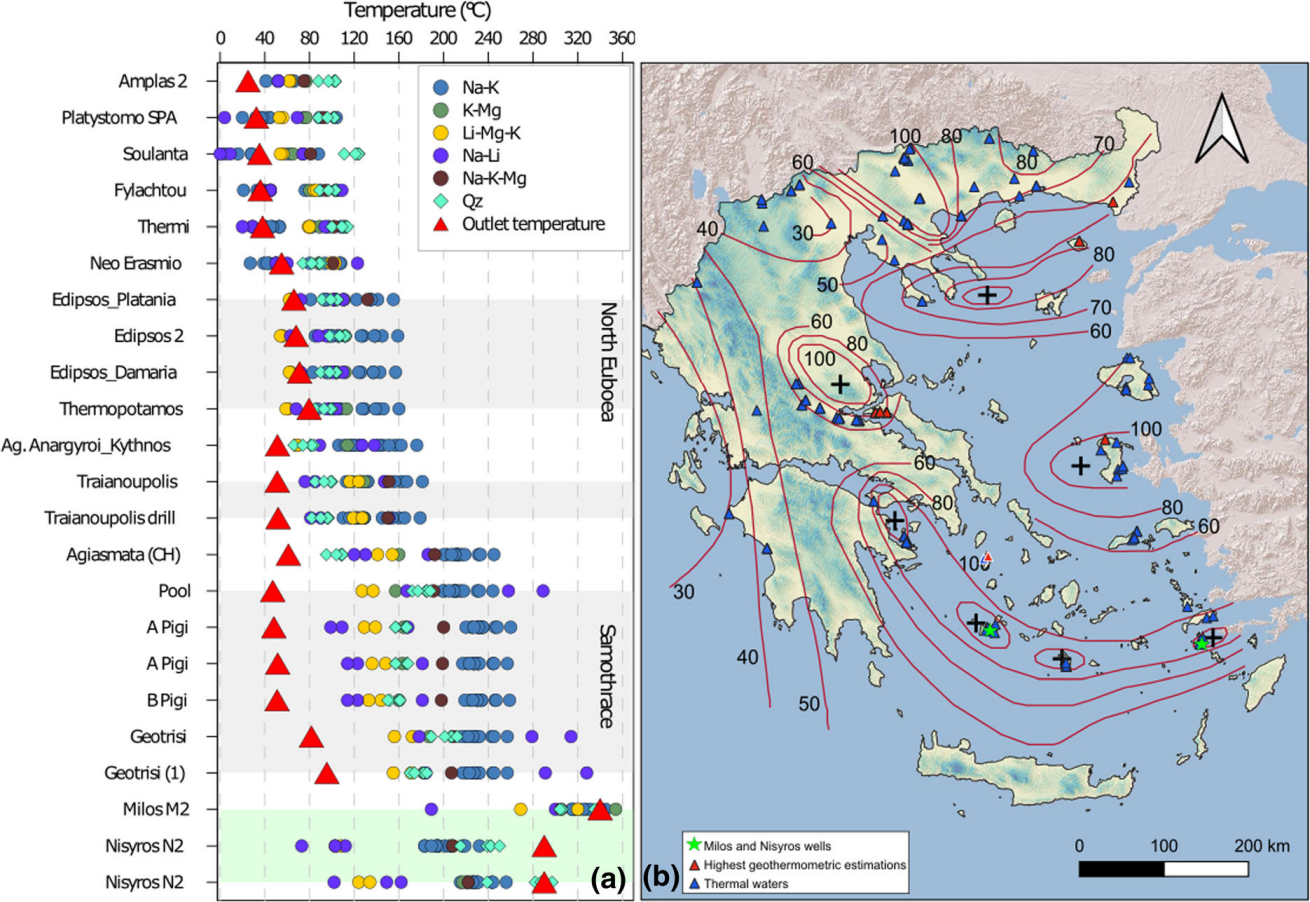
**a** Results of the geothermometric estimations made with the computer program SolGeo (Verma et al., 2008) on selected samples that plotted in the partial equilibration field of the Giggenbach diagram of Fig. 9. **b** Thermal waters plotted on heat flow map (mW/m^2^) (Fytikas & Kolios, 1979); red triangular indicates the highest estimated temperatures from geothermometric equations

Many of the partially equilibrated samples are close to the seawater point, and thus, the temperature of the reservoir could not be estimated due to the contamination with marine water. Only a few partially equilibrated samples mostly from Samothraki Island and Edipsos area may be considered reliable indicating temperatures up to 240–260 °C (Geotrisi). All these sites were selected and compared to the temperatures obtained from other geothermometric equations using the software Solute Geothermometers (SolGeo—Verma et al., 2008), which estimates the minimum temperature of the aquifer by comparing thirty-five geothermometric equations (Fig. 10a). The geothermometric estimations made with this software are based on the solute concentrations of the sampled waters. Many empirical geothermometric equations have been proposed by different researchers during the years and have been grouped together in this program. Most of them are based on cationic contents of the waters: 13 equations are based on the Na^+^ and K^+^ contents of the waters, 3 on K^+^ and Mg^2+^, 2 on Li^+^ and Mg^2+^, 5 on Na^+^ and Li^+^, 3 on Na^+^, K^+^ and Ca^2+^, 1 on Na^+^, K^+^ and Mg^2+^, 2 on Na^+^, K^+^, Ca^2+^ and Mg^2+^ and finally 7 are silica geothermometers based on the SiO_2_ content. For the references of these geothermometers, we refer to the paper of Verma et al. (2008) while the formulas are included as supplementary material (Table S2).

As generally happens, the equations do not give back a unique temperature, but a wide range. The Na–K geothermometers show mostly the highest temperatures of reservoirs, with up to about 40 °C differences. On the contrary, Li-Mg–K and Na-Li geothermometers present the lowest results; sometimes below the outlet temperature of the spring. It should be mentioned that the Na–K geothermometers are considered the most reliable because less affected by mixing with shallow waters and degassing (Pope et al., 1987).

Excluding geothermometers giving estimations at or below the emergence temperature, the narrowest range is shown at thermal waters with estimated temperatures below 120 °C (Fig. 10a) and comprise samples from central Greece (Soulanta, Platystomo SPA and Amplas2) and from northeastern Greece (Thermi, Neo Erasmio and Fylachtou).

A few sites gave Na–K estimations up to 160–180 °C (Fig. 10a) indicating an important geothermal potential for the area of Edipsos (Northern Evia Island), Traianoupolis (Thrace) and Agioi Anargyroi (Kythnos Island). Only two areas gave estimations above 200 °C and up to 250 °C: the areas of Therma on Samothraki Island and of Agiasmata on Chios Island. For these systems, further studies should ascertain if they might be used also for electricity production.

To support this hypothesis, Fig. 10b evidences a correspondence among thermal springs and heat flux. The majority of studied thermal springs are located in areas with heat flux higher of 60 mW/m^2^, while the highest values of temperature obtained from geothermometric estimation correspond to areas with elevated heat flow (> 80 mW/m^2^). The maxima of heat flux (> 120 mW/m^2^), found along the SAAVA, do not show thermal springs with high measured temperature or high geothermometric estimates. This does not mean that no high-temperature geothermal system is present, but only that its impermeable seal is very efficient. As previously mentioned, in fact, two high-enthalpy geothermal systems have been ascertained on the islands of Milos and Nisyros by explorative drillings. The geothermometric estimates made with the composition of the captured waters gave results closely resembling the measured temperature in the case of Milos (Fig. 10a). In the case of Nisyros, the close correspondence between estimation and measured value is found only for the silica geothermometer (Fig. 10a), which is less influenced by the contamination of the seawater used for the drilling mud (Chiodini et al., 1993).

### Tectonic structures as a fluid carrier

After studying the gas emissions of Greece, Daskalopoulou et al. (2018a) noticed a connection between lithological facies and dominant gas components. Particularly, the sedimentary regime of EH, where hydrocarbon deposits are present, yield that CH_4_ and N_2_ are the dominant gas species in the area. On the other hand, CO_2_ is the principal gas component in IH and HH, where intrusive and metamorphic formations prevail. On the basis of the geographical distribution of the gases, a similar behaviour between CO_2_ concentrations and *R*_C_/*R*_A_ ratios is noticed (Daskalopoulou et al., 2018a, 2019a). This reflects increasing CO_2_ and *R*_C_/*R*_A_ values in areas characterised by thin crust, elevated heat flow values, Plio-Quaternary volcanic activity and deep routed extensional or transtensional regional faults (Fig. 2b) (Daskalopoulou et al., 2019a); VA displays a higher mantle contribution for both CO_2_ and He with respect to EH that present an important crustal input (Daskalopoulou et al., 2019a).

As faults create a permeable pathway for gases to ascend (Wang & Jaffe, 2004), special attention was given to places with complex tectonics. In northern Greece, the Strymonas Fault System is controlling the tectonics. In this area, gases collected from thermal waters, present an up to 15% mantle contribution for He (considering a MORB end-member). This is likely caused due to the U- and Th-rich minerals (i.e. zircon, apatite etc.—Wüthrich, 2009) of the granitoids present in this area; the decay of U and Th produces ^4^He thereafter depleting the R/R_A_ values. Gases collected from the thermal waters of Euboea and Samothraki are found in the Grecian Shear Zone (propagation of the NAF towards the Hellenic mainland—Şengör, 1979). According to various authors (Güleҫ & Hilton, 2006; Güleҫ et al., 2002; Mutlu et al., 2008), almost more than 50% of the He along some sections NAF derives from the mantle, hence *R*/*R*_A_ values are expected to be enriched in mantle He. Gases collected from the cold waters of Corinth Gulf are generally dominated by CH_4_ and N_2_ and the associated He shows always *R*/*R*_A_ values typical to pure crustal origin (Pik & Marty, 2009). The same authors explained that the fault system is not connected in depth with zones in which mantle He can be trapped or that reaches the lower crust allowing the uprise of mantle fluids. It is worth mentioning that in the western part of the graben system (Saronikos gulf), where it meets the SAAVA, the Quaternary volcanic activity of Sousaki and Methana allowed CO_2_-rich fluids to reach the surface.

The above described extensional and transtensional deep regional tectonic structures, which are permeable pathways to the earth’s surface for mantle or deep crustal fluids, allow also deep circulation of groundwater creating the conditions for the formation of small hydrothermal systems. In the case of the presence of an important heat source, along such tectonic structures some greater or higher temperature hydrothermal systems may form. This is the case of both Samothraki island and Edipsos (Fig. 10b) where the heat source is a Ternary granitic intrusion in the former case (Dotsika, 2012) and a Quaternary volcanic system in the latter (D’Alessandro et al. 2014).

## Conclusions

Thermo-mineral waters in Greece are strongly controlled by the geologic and geodynamic setting that characterises the area where they are found. On the basis of pH, hyperalkaline Ca–OH-type waters were identified in the ophiolitic sequences of Argolida and Othrys, while acidic waters were documented on islands located along the SAAVA. The former group represents the evolution of waters in deep serpentinised ultramafic aquifers in conditions closed to the atmosphere, while the latter highlights the impact of the volcanic/geothermal degassing on the waters. Based on their temperature, the remaining samples were subdivided into cold and thermal. Cold waters found in the northern part of Greece showed high *p*CO_2_ values and were characterised by Ca–HCO_3_ composition. Carbon dioxide dissolution resulted in slightly acidic waters with elevated bicarbonate content. Saturation or oversaturation in the carbonate minerals is not common in this group. Few water samples with intermediate *p*CO_2_ were saturated, while the lack of equilibrium with carbonate minerals underscored the importance of CO_2_ dissolution. Cold waters collected in hydrocarbon-prone areas of western Greece presented low *p*CO_2_ and were close to equilibrium with the main carbonate minerals suggesting that the petrological regime (sedimentary limestones) governs the equilibrium of the dissolved carbonate species. On the other hand, thermal waters were seemingly controlled by the high heat flow values and the low crustal thickness. Their chemical composition was strongly influenced by mixing processes between meteoric water and seawater. Geothermometry was applicable only in few partially equilibrated waters suggesting reservoir temperatures from 80 to 260 °C with the most elevated values (between 200 and 260 °C) being found in two islands of the eastern Aegean Sea (Samothraki and Chios).

This extensive dataset represents an almost complete catalogue of hydrogeochemical data on thermo-mineral waters of the whole Greece. This dataset has been gathered for over 15 years by the same research group and analysed in the same laboratory ensuring a good analytical uniformity. This database represents therefore a good basis for future studies on the thermo-mineral waters of this country.

## Supplementary Information

Below is the link to the electronic supplementary material.

Supplementary file1 (XLSX 176 kb)

Supplementary file2 (XLSX 23 kb)

## Data Availability

All data are included in the supplementary material as Excel spread sheet.
